# IDENTIFYING DYAD-LEVEL PARENTING PRACTICES AMONG US CUSTODIAL GRANDPARENTS: A LATENT PROFILE ANALYSIS

**DOI:** 10.1093/geroni/igad104.0403

**Published:** 2023-12-21

**Authors:** Minzhi Ye

**Affiliations:** Kent State University, Kent, Ohio, United States

## Abstract

Although half of US custodial grand families contain married grandparents, little is known about the parenting practices of custodial grandfathers (CGF). Based on the Family Stress model and the latent profile analyses, we identified typologies of dyad-level parenting practices among 193 married custodial grandparents (CGP) couples recruited nationally through convenience-based and population-based strategies. Grandmothers (CGM) and CGF were interviewed separately, and four types of parenting practices profiles emerged: (1) Ideal CGM&CGF; (2) Inadequate CGM & Ideal CGF; (3) Ambivalent CGM & High-effective CGF; and (4) High-effective CGM & Inadequate CGF. After controlling for socio-demographics, these profiles related differentially to the psychological well-being of CGF and CGM, as well as to the behavioral and emotional outcomes of grandchildren (CGC). CGF in Profile four reported significantly lower positive affect than those in Profiles one and three, but there were no profile differences regarding CGF depressive symptoms. While CGM in Profile two reported fewer depressive symptoms than the other profiles, there were no differences regarding GM’s positive affect. A pattern also occurred whereby CGC from Profile one had significantly fewer externalizing and internalizing difficulties than other profiles. It is essential for future researchers and practitioners to recognize the contribution of CGF to the parenting dynamics in families with married CGP.

